# Syncope: Atypical Presentation of Diaphragmatic Hernia

**DOI:** 10.7759/cureus.51715

**Published:** 2024-01-05

**Authors:** Hazem AlHazmi, Ammar Y Bahadur, Khalid AlAhmadi, Ola Y Bahadur

**Affiliations:** 1 Pediatric Emergency Medicine, King Abdulaziz Medical City Jeddah, Jeddah, SAU; 2 Pediatrics, King Abdulaziz Medical City Jeddah, Jeddah, SAU; 3 Medicine and Surgery, King Saud Bin Abdulaziz University for Health Sciences College of Medicine, Jeddah, SAU

**Keywords:** diaphragmatic hernias, congenital diaphragmatic hernia (cdh), pediatrics, emergency, syncope

## Abstract

In this case report, we describe a rare presentation of diaphragmatic hernia in a pediatric patient presenting with syncope. Congenital diaphragmatic hernia (CDH) is a developmental discontinuity of the diaphragm that causes the abdominal viscera to herniate into the thoracic cavity. It is usually diagnosed shortly after birth and is often associated with pulmonary hypoplasia and pulmonary hypertension, causing life-threatening conditions, or it could be completely asymptomatic. Syncope is induced by various conditions such as cerebrovascular disease, arrhythmia, hypoglycemia, anemia, epilepsy, and autonomic nervous disorder.

## Introduction

Congenital diaphragmatic hernia (CDH) is a developmental disorder that predominantly manifests as respiratory distress in neonates within the first few hours of life. This respiratory distress can range from mild to severe and even life-threatening [1]. However, it is quite unusual for CDH to develop beyond the neonatal period. Late-presenting CDH presents a challenge for clinical diagnosis because it can be associated with various symptoms [1]. Here, we describe a rare case of CDH in a seven-year-old child who presented with syncope.

## Case presentation

A seven-year-old child with sensorineural hearing loss, who is otherwise in good health and uses a hearing aid, arrived at our emergency room (ER) after losing consciousness at school. The child mentioned that she experienced lightheadedness during class, followed by a loss of consciousness and collapsing. We contacted her teacher, who reported finding the child not breathing and administered rescue breaths. They also attempted to stimulate the child by shaking her and calling her name. The child regained consciousness after approximately one minute. Following this incident, she left the school with her mother and came to our ER for further investigation.

This episode occurred suddenly without any preceding symptoms such as dizziness, aura, vertigo, tinnitus, or ear pain. There was no history of trauma, and the episode was unrelated to physical exertion. During the event, there were no abnormal movements, loss of sphincter control, or eye-rolling, nor was there a clear postictal state. The child mentioned that, for the two days prior to the episode, she had experienced upper respiratory tract symptoms (coughing and a runny nose), which were managed with normal saline nasal drops and acetaminophen. Both the child and her mother denied any history of fever, increased breathing difficulties, apnea, or cyanosis. There was no report of palpitations, chest pain, or excessive sweating.

The child had been maintaining her regular diet with no vomiting, diarrhea, or changes in bowel habits. She denied dysuria and changes in urine color or odor and had never experienced similar episodes before. She is up-to-date with her vaccinations, and her family history includes an 18-year-old brother who also uses hearing aids. The child is not on any medications, has no known allergies, and has never been admitted to the hospital or undergone surgery. She was born full-term and did not require neonatal intensive care unit (NICU) admission.

Upon examination in the ER, the child appeared well, conscious, active, and communicative. She exhibited a good complexion, was well-hydrated, had stable vital signs, and maintained satisfactory oxygen saturation above 97% on room air without signs of distress. Her vital signs were as follows: BP 105/96 mmHg within the 90th centile for her age and sex, a heart rate (HR) of 120 beats per minute, a respiratory rate (RR) of 30 breaths per minute, and a temperature of 37.2 °C. Her weight was 16.3 kg, and her height was 116 cm. She was alert and cooperative during the examination.

The head and neck examination revealed no abnormalities, and her pupils reacted bilaterally. The ear, nose, and throat (ENT) examination showed a congested throat with no follicles and clear tympanic membranes. Her lungs were clear upon auscultation. Cardiac examination indicated a regular rate and rhythm with normal S1 and S2 heart sounds, with no murmurs or gallops detected. Perfusion was adequate, and capillary refill was one second at her fingertip. Her abdomen was soft, with no organomegaly or palpable masses. The skin examination showed no remarkable findings.

Initial laboratory results included a complete blood count, which showed a hemoglobin level of 8.8 g/dL, a white blood cell count of 22,000/mm^3^, and a platelet count of 477/mm^3^. Electrolyte levels were within the normal range. Kidney function tests showed a creatinine level of 53 mmol/L and a BUN of 4.4 mmol/L. Arterial blood gas analysis revealed a pH of 7.34, pCO_2_ of 40.6, pO_2_ of 91.1, and HCO_3_ of 21.6.

**Figure 1:**
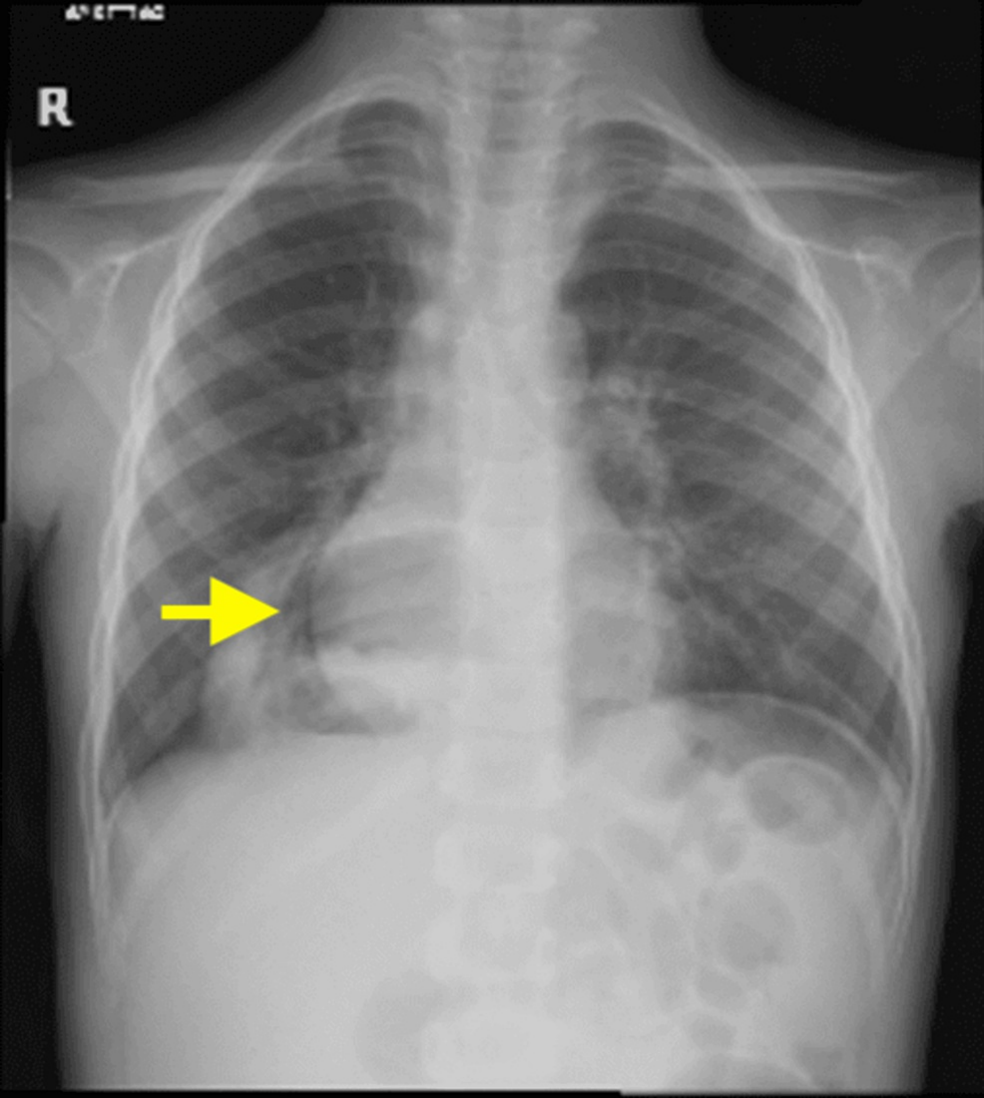
Retrocardiac lucent tubular shadows with air-fluid level; chest CT was then performed to rule out diaphragmatic hernia

**Figure 2:**
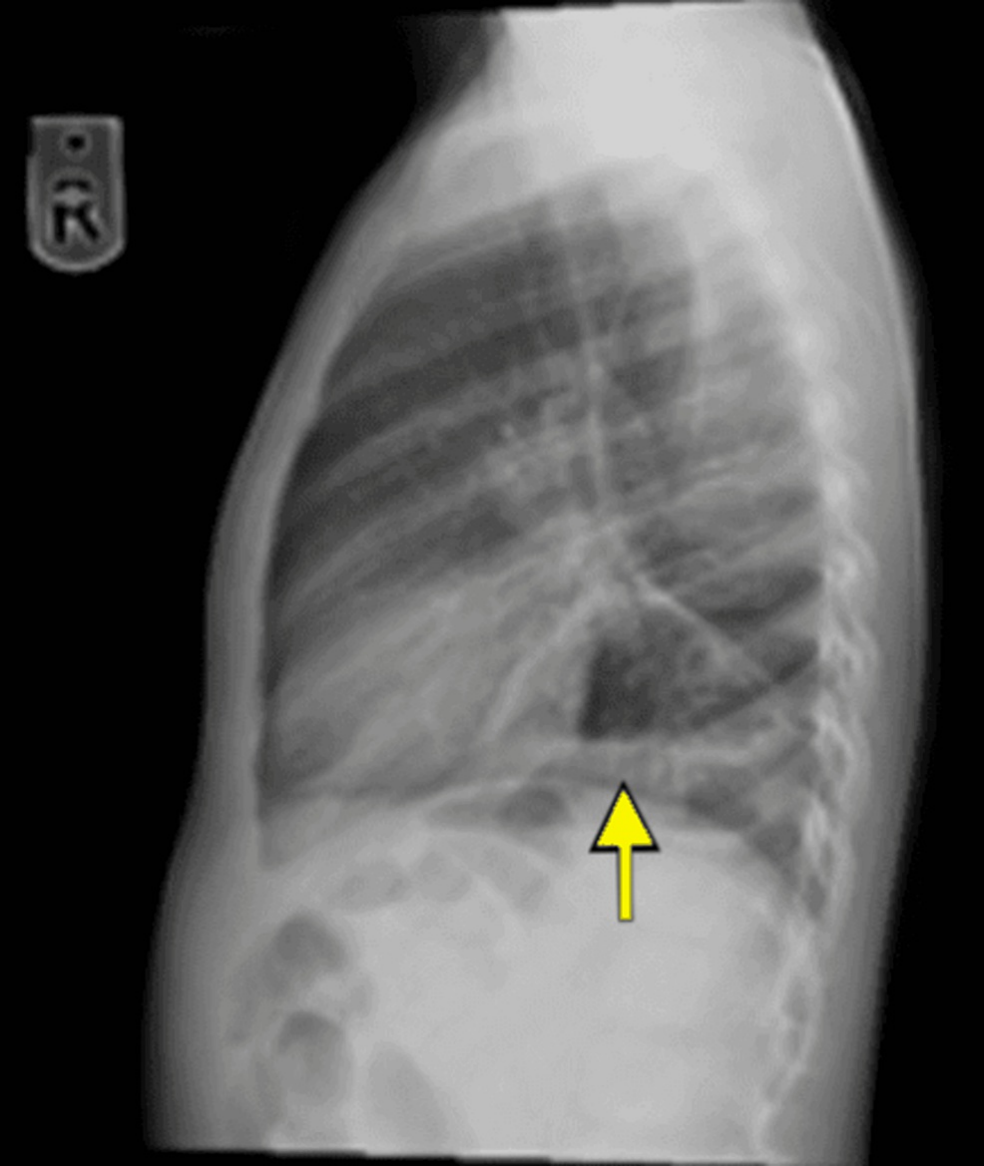
Retrocardiac lucent tubular shadows with air-fluid level; chest CT was then performed to rule out diaphragmatic hernia

The electrocardiogram (ECG) displayed sinus tachycardia with a heart rate of 116 and a normal QT interval of 337 ms. Chest X-rays (Figures 1-2) revealed retrocardiac lucent tubular shadows with air-fluid levels, prompting further investigation with CT chest scans to rule out diaphragmatic hernia. Additionally, there was a pericardiac air space opacity in the lower right lung zone, although it was unlikely to indicate compressive atelectatic changes. There were no observed pleural effusion or pneumothorax, and the cardio-mediastinal contour appeared within normal limits. There was no evidence of aggressive osseous lesions.

**Figure 3:**
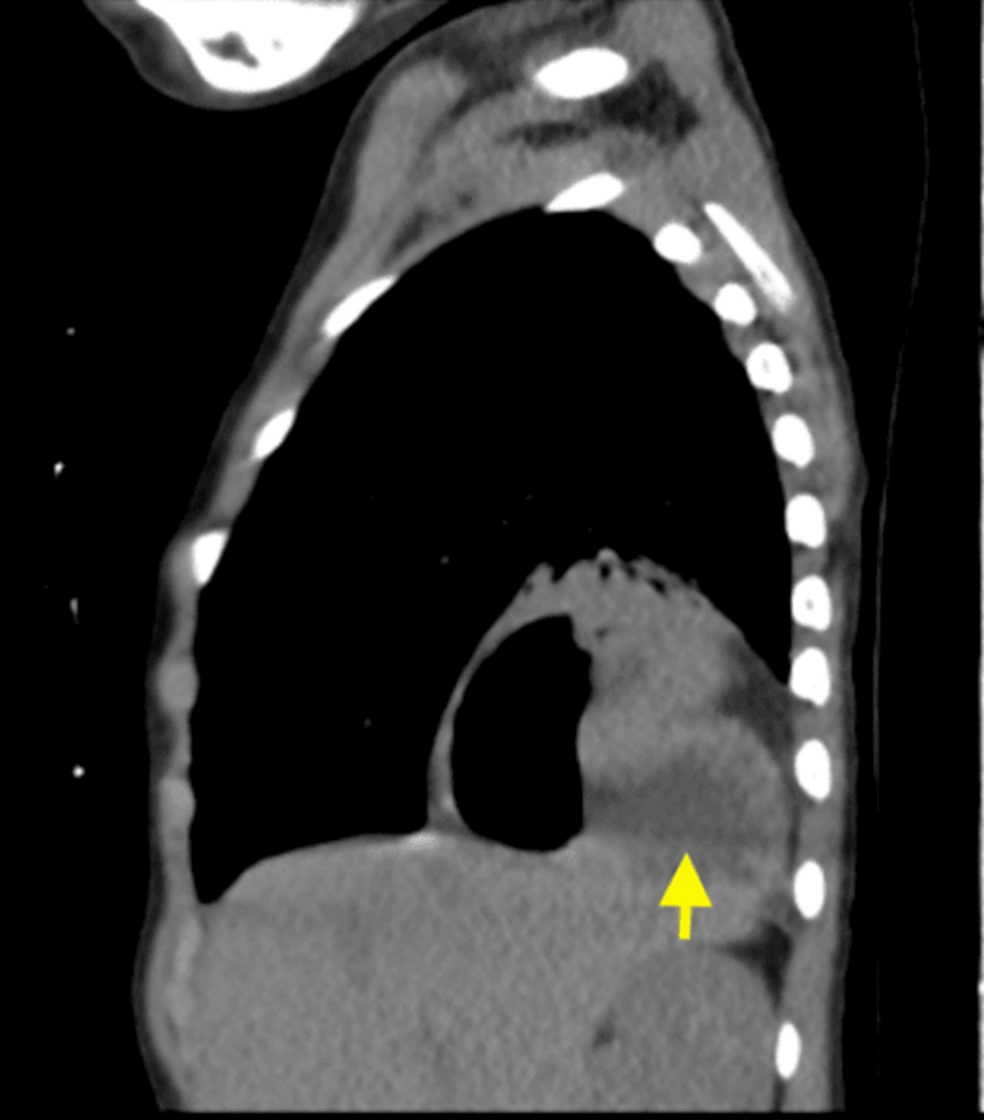
CT chest showed right posterior medial diaphragmatic defect with small bowel and stomach seen herniating into the chest (right diaphragmatic hernia)

**Figure 4:**
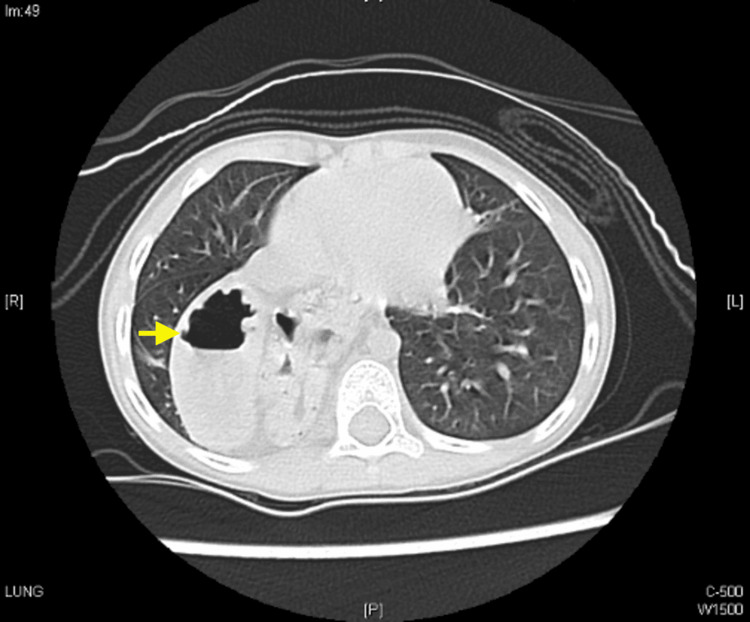
CT chest showed right posterior medial diaphragmatic defect with small bowel and stomach seen herniating into the chest (right diaphragmatic hernia)

The CT chest showed a right posterior medial diaphragmatic defect, with the small bowel and stomach seen herniating into the chest (right diaphragmatic hernia) (Figures 3-4).

The patient was admitted to the pediatric ICU for close observation to rule out any potential cardiopulmonary complications. During the patient’s stay, a neurology assessment and EEG were conducted, which ruled out neurological causes of the syncope. Cardiology specialists were also consulted for a comprehensive evaluation. At that point, the patient had been started on a beta-blocker regimen. Various cardiac tests were performed, including a cardiac electrophysiology study, 24-hour Holter monitoring, echocardiography, and an exercise stress test, all of which showed no evidence of prolonged QT syndrome, arrhythmias, or any other anatomical abnormalities.

**Figure 5:**
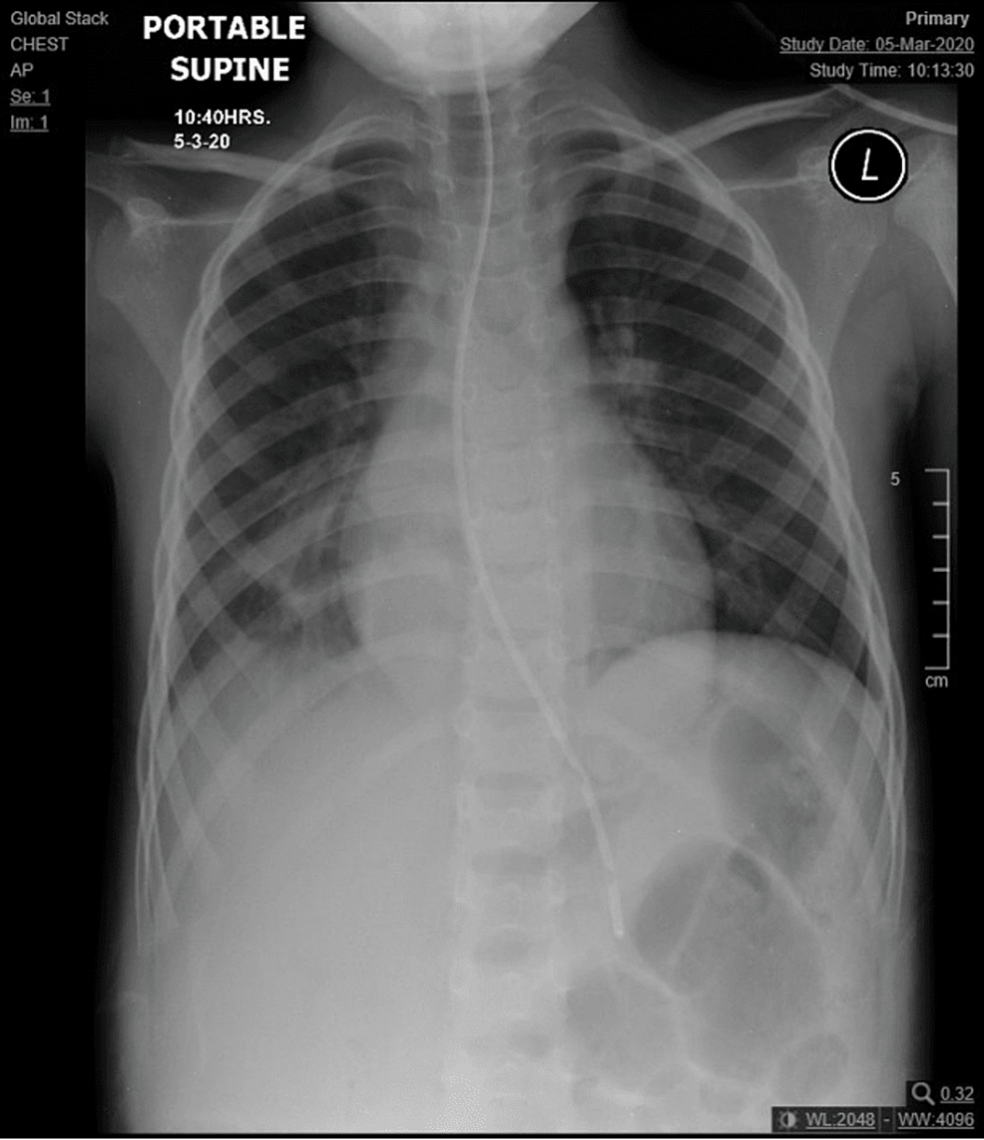
Post-operatively the chest X-ray

Subsequently, the beta-blocker treatment was discontinued, and the patient was given clearance by the cardiology team for diaphragmatic hernia repair. The patient was discharged and readmitted three weeks later for this procedure. Following the surgery, a post-operative chest X-ray was conducted (Figure 5), and the patient remained clinically and vitally stable without any post-operative complications. The patient was discharged home with scheduled follow-ups involving pediatric surgery, pediatric cardiology, and genetics.

**Figure 6:**
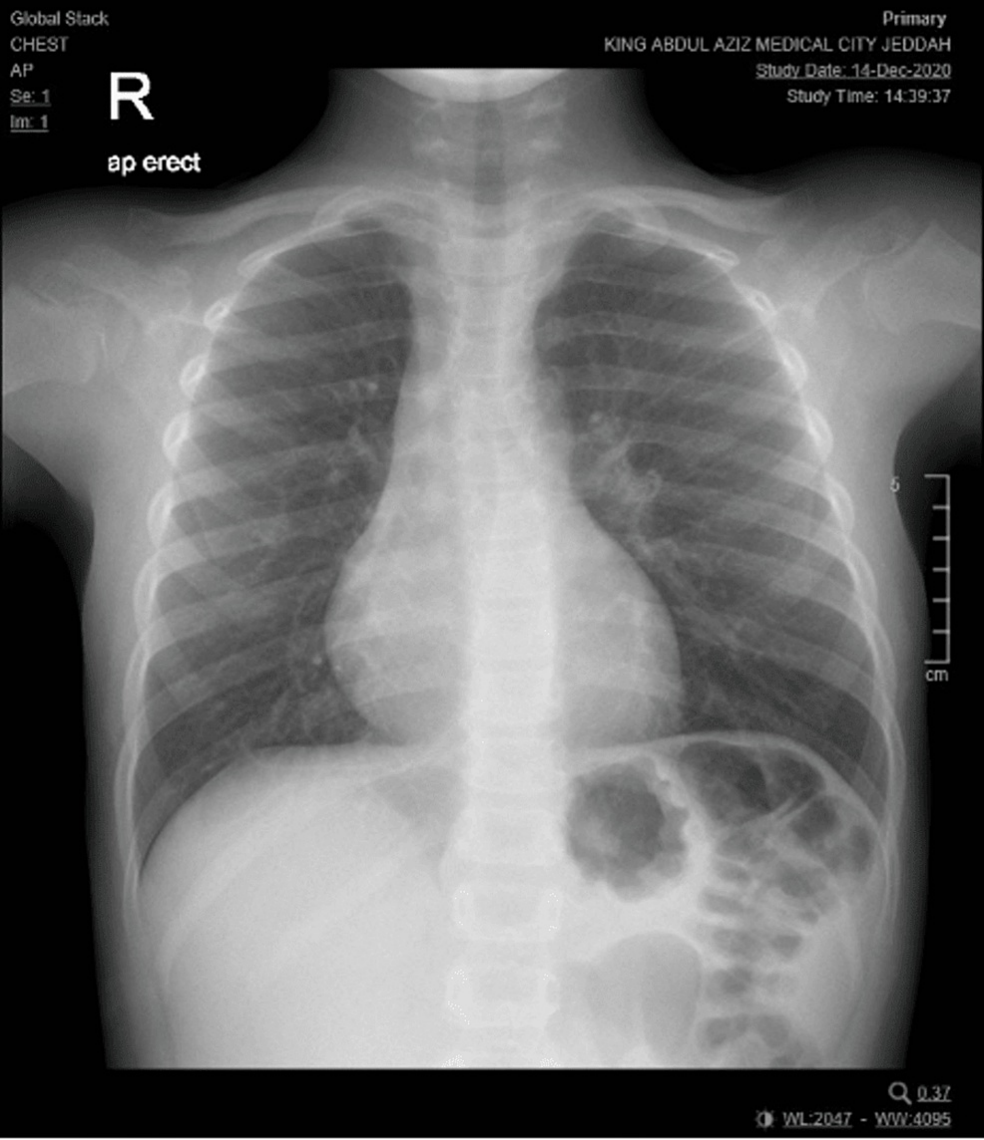
X-ray repeated at clinic

Genetic testing, specifically whole-exome sequencing, yielded negative results, leading to the patient’s discharge from the genetics department. During the last follow-up with pediatric surgery in December 2020, a chest X-ray was repeated, and no abnormalities were observed. The child continued to grow and thrive without any concerns or recurrence of the syncope and was subsequently discharged from the clinic (Figure 6).

## Discussion

CDH is a developmental abnormality of the diaphragm, resulting in abdominal organs protruding into the thoracic cavity. Typically, CDH is detected shortly after birth and is often associated with life-threatening conditions like pulmonary hypoplasia and pulmonary hypertension [1]. CDH is typically diagnosed within the first few weeks or months after birth. However, in approximately one in four cases, CDH is not identified until adulthood [2].

CDH can manifest in various ways, ranging from asymptomatic cases to symptoms resembling intestinal obstruction or acute respiratory failure. Pediatric patients may also experience respiratory symptoms such as coughing and respiratory distress. Occasionally, CDH is incidentally discovered during the evaluation or treatment of other medical conditions [3].

Syncope, or fainting, can be triggered by various factors, such as cerebrovascular disease, arrhythmias, hypoglycemia, anemia, epilepsy, and autonomic nervous system disorders. Moreover, in a few cases, syncope can result from swallowing, a reflex mediated by the vagus nerve. Underlying conditions such as esophageal disorders (e.g., achalasia, diffuse esophageal spasm, hiatal hernia, and diverticulum) and specific food and drink items (e.g., cold water, hot liquids, or carbonated beverages) have been reported to induce syncopal episodes. Most cases of swallow-induced syncope are associated with bradycardia, atrioventricular block, or ventricular arrhythmias [4].

In this case, the medical team suspects that the patient may have experienced a phenomenon known as the “swallow syncope” syndrome. This syndrome has been recognized for several decades, although its exact cause remains uncertain. One hypothesis suggests that syncope in these cases may result from sinus or nodal bradycardia, sinus arrest, or an advanced atrioventricular block. Alternatively, it is possible that the mechanical pressure exerted by a large hiatal hernia on the left atrium, especially after a heavy meal or while in a recumbent position, may lead to reduced cardiac output and, ultimately, syncope [5,6].

While diaphragmatic hernias can be suspected from chest X-rays, a definitive diagnosis of hiatal hernia requires confirmation through other imaging techniques such as barium swallow, computed tomography, or, preferably, cardiac magnetic resonance(CMR) imaging to avoid radiation exposure [5]. Regardless of how CDH is initially presented, surgery is recommended because of the risk of organ incarceration [3]. In most cases, a laparoscopic approach is preferred [3]. The hernia repair can be performed using various techniques, including primary closure, the use of mesh, or total excision of the hernia sac [3]. In situations where surgical intervention and direct correction of the defect are not feasible, an alternative approach involves managing the symptoms through dietary modifications. Specifically, individuals with CDH may benefit from consuming smaller meal sizes, as this can help alleviate some of the associated symptoms [6].

## Conclusions

CDH is a rare defect that may present with various symptoms, such as syncope or signs of intestinal obstruction, or may be discovered incidentally. Syncope associated with congenital diaphragmatic hernia in a school-aged child with sensorineural hearing loss is a rare condition that warrants further investigation. As surgical repair was successful, early detection could carry a favorable outcome.
